# Supplementary material for: 3D cell segregation geometry and dynamics are governed by tissue surface tension regulation
Source: Commun Biol. 2023 Aug 4;6:817. doi: 10.1038/s42003-023-05181-7 (PMC10403547; doi:10.1038/s42003-023-05181-7)
Supplement: Supplementary file 2 — Supplementary Information [file 42003_2023_5181_MOESM2_ESM.pdf]

3D cell segregation geometry and dynamics are governed by tissue surface tension regulation

Elod Méhes, Enys Mones, Máté Varga, Áron Zsigmond, Beáta Biri-Kovács, László Nyitray, Vanessa Barone, Gabriel Krens, Carl-Philipp Heisenberg, and Tamás Vicsek

Supplementary Figure 1

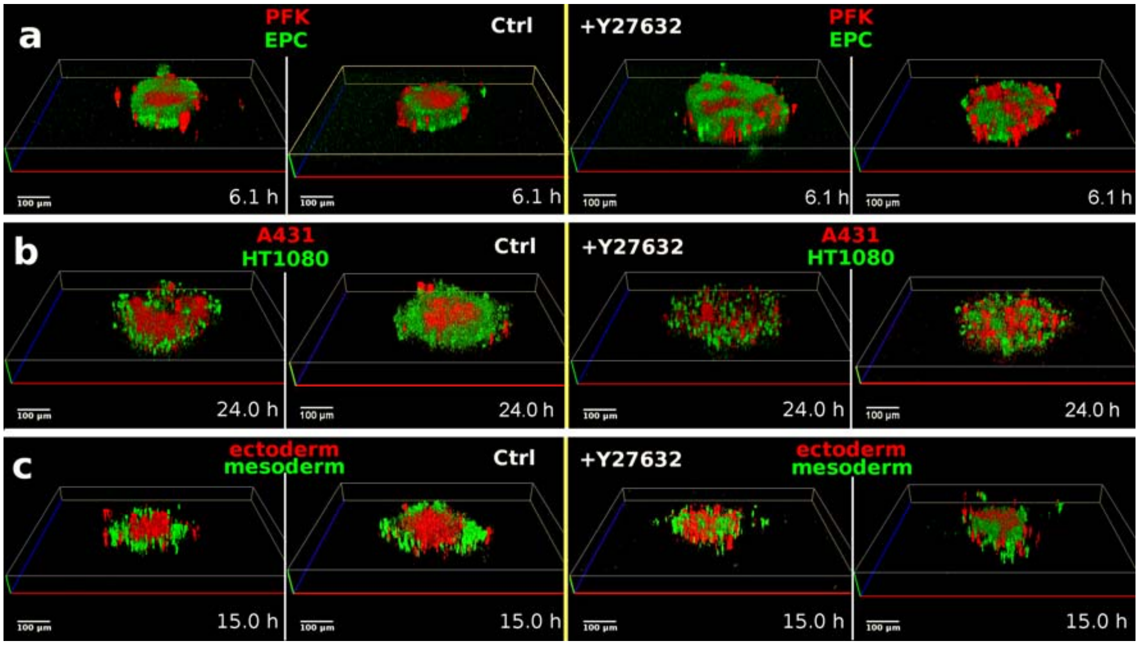

3D segregation is delayed by general pharmacological inhibition of actomyosin contractility.

a) Representative 3D reconstruction images from time-lapse videos of segregating clusters of PFK (red) and EPC (green) keratinocytes 6 h after start of the segregation process in absence (left panels) or presence (right panels) of 100 µM Y27632 ROCK inhibitor.

b) Images of A431 epithelial carcinoma (red) and HT1080 fibrosarcoma (green) segregation in absence (left) or presence (right) of 50 µM Y27632, after 24 h segregation. Note the formation and delayed fusion of multiple clusters of A431 cells inside the spheroids under ROCK inhibition (right).

c) Representative images of zebrafish ectoderm (red) and mesoderm (green) segregation in absence (left) or presence (right) of 100 µM Y27632, after 15 h segregation.

Time elapsed after initial mixing of heterotypic cell suspensions is indicated at the lower right corner of each image, scale bars: 100 µm. The vertical yellow line indicates the border between left and right panels.

Supplementary Figure 2

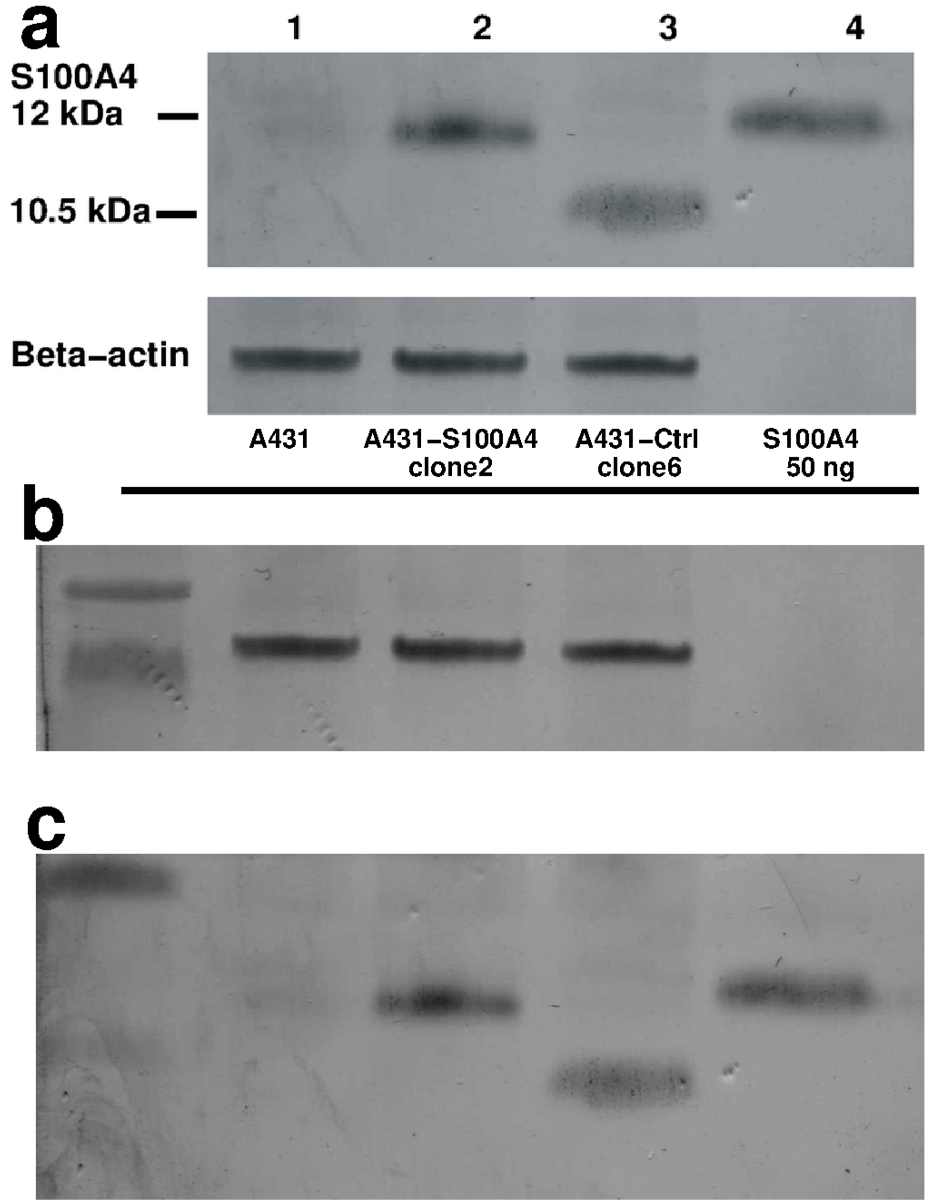

Western blot detecting the S100A4 isoforms in A431 human epithelial carcinoma clones.

a) S100A4 protein was detected in immunoblots of A431 clones (upper panel). Lysates from A431 cells (lane 1: A431), and A431 clones overexpressing either wild type S100A4 (lane 2: A431-S100A4), or non-functional truncated mutant S100A4 (lane 3: A431-Ctrl) are shown with a 50 ng standard recombinant wild type S100A4 protein sample for comparison as positive control (lane 4). Wild type S100A4 has a relative molecular mass of 12 kDa while truncated mutant S100A4 is 10.5 kDa, indicated on left side. Lower panel: For loading control, the upper part of the blot was immunolabeled for beta-actin (42 kDa). **b,c**) Unedited images of the blot cut into upper and lower parts and developed separately, molecular mass markers are seen in the left lane. **b**) Upper part of the blot developed with anti-beta-actin antibody.

c) Lower part of the blot developed with anti-S100A4 antibody.

Supplementary Figure 3

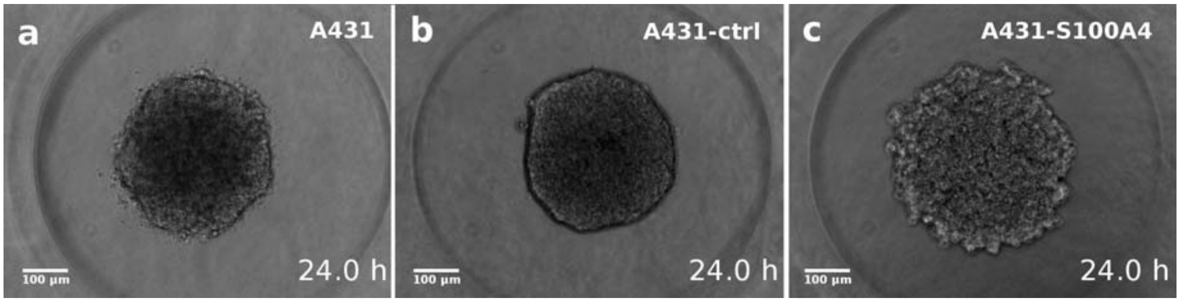

Aggregation morphology depends on non-muscle myosin 2 assembly and function.

a) Representative phase-contrast image from a time-lapse video of normal A431 cells forming a spheroid after 24 h of aggregation from suspension.

b) Image of a spheroid formed of A431 cells overexpressing the inactive mutant isoform of S100A4 (A431-ctrl). Note the smooth surface of the compact spheroids in a) and b) panels.

c) Representative image of a spheroid formed of A431 cells overexpressing the NM2 assembly inhibitor S100A4 (A431-S100A4). Note the berry-like surface and less compact morphology of the spheroid in c).

Time elapsed after start of aggregation from cell suspension is indicated at the lower right corner of each panel image, scale bar: 100 µm.

Supplementary Figure 4

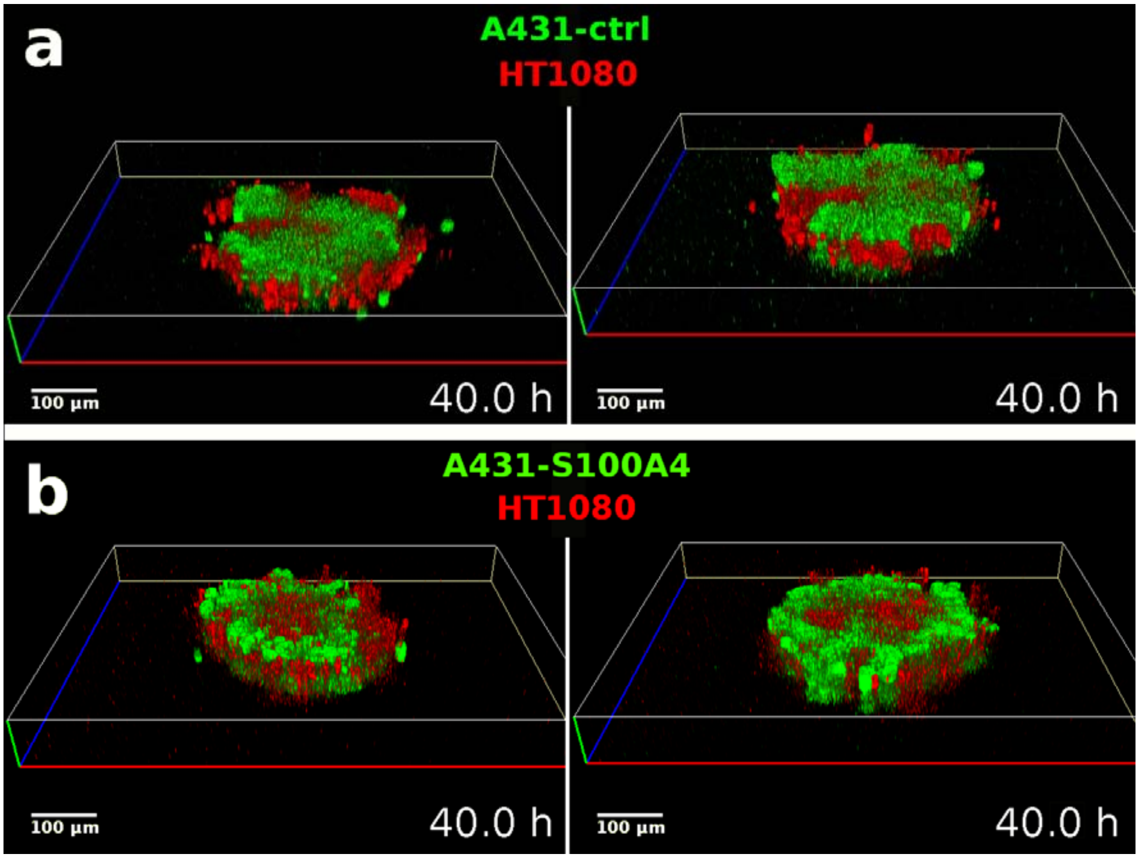

Spatial positioning during segregation depends on non-muscle myosin 2 assembly and function.

a) Representative 3D reconstruction images from time-lapse videos of HT1080 fibrosarcoma cells (red) segregating from A431-ctrl epithelial carcinoma cells (green) overexpressing the inactive mutant S100A4 isoform. Note the segregation of A431-ctrl (green) clusters to the inside of spheroids.

b) Representative 3D images of segregation of HT1080 cells (red) from A431-S100A4 cells (green) overexpressing the NM2 assembly inhibitor S100A4. Note the tendency of A431-S100A4 (green) clusters to segregate to the periphery of spheroids.

Time elapsed after initial mixing of heterotypic cell suspensions is indicated at the lower right corner of each image, scale bars: 100 µm.

Supplementary Figure 5

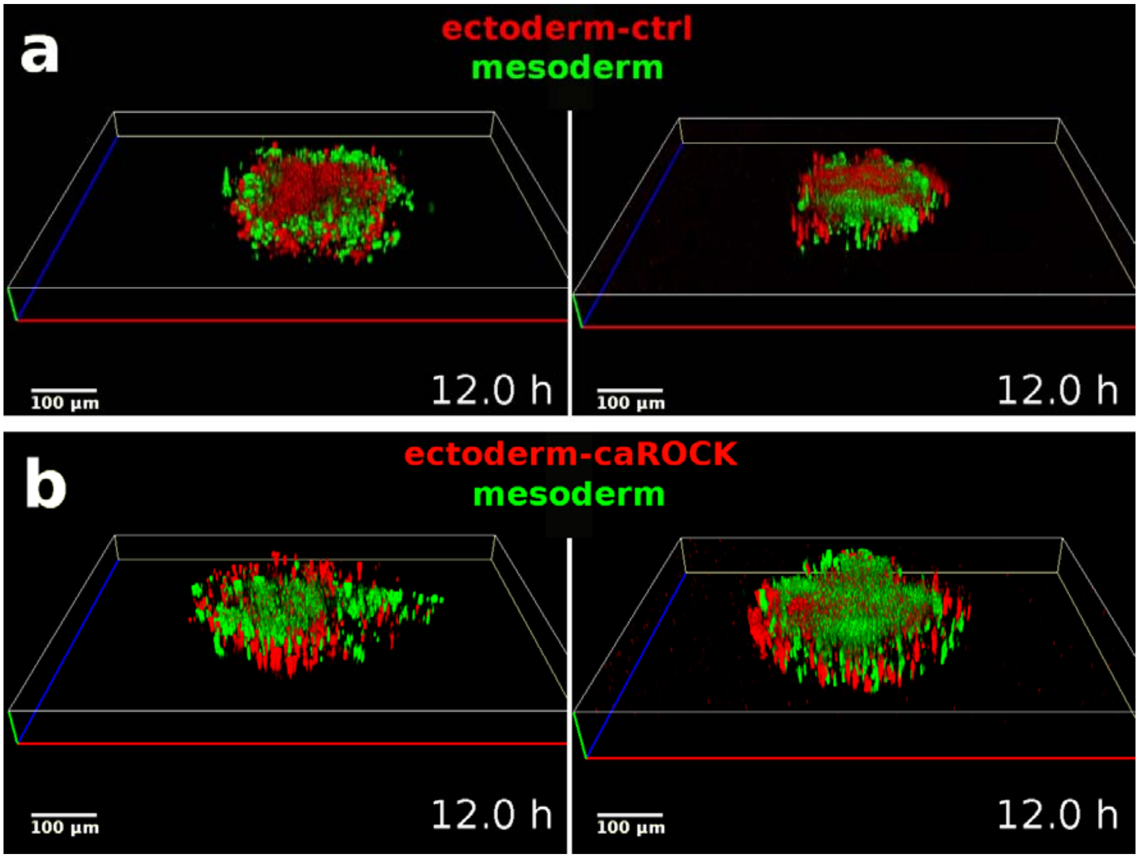

Spatial configuration of segregated domains depends on actomyosin contractility regulation.

a) Representative 3D reconstruction images from time-lapse videos of mesoderm cells (green) segregating from normal ectoderms cells (red) isolated from zebrafish embryos. Note the segregation of ectoderm (red) clusters to the inside of spheroids.

b) Representative 3D images of segregation of mesoderm cells (green) from ectoderm-caROCK cells (red) overexpressing constitutively active ROCK. Note the tendency of ectoderm-caROCK (red) clusters to segregate to the periphery of spheroids.

Time elapsed after initial mixing of heterotypic cell suspensions is indicated at the lower right corner of each image, scale bars: 100 µm.
