# Supplementary material for: 3D cell segregation geometry and dynamics are governed by tissue surface tension regulation
Source: Commun Biol. 2023 Aug 4;6:817. doi: 10.1038/s42003-023-05181-7 (PMC10403547; doi:10.1038/s42003-023-05181-7)
Supplement: Supplementary file 3 — Description of Additional Supplementary Files [file 42003_2023_5181_MOESM3_ESM.pdf]

Description of Additional Supplementary Files

File name: Supplementary Movie 1
Description: Separation force measurement by Dual Pipette Aspiration method. Separation of primary goldfish keratinocyte (PFK) doublet, real time brightfield microscopic imaging.

File name: Supplementary Movie 2
Description: Separation force measurement by Dual Pipette Aspiration method. Separation of EPC fish keratinocyte doublet, real time brightfield microscopic imaging.

File name: Supplementary Movie 3
Description: Basic segregation of primary goldfish keratinocytes (PFK, red) and EPC(green) fish keratinocyte cell line in suspension. Epifluorescent microscopy time-lapse video recorded at 10 min/frame rate.

File name: Supplementary Movie 4
Description: Basic segregation of A431 human epithelial carcinoma (red) and HT1080 human fibrosarcoma (green) cells in suspension. Epifluorescent microscopy time-lapse video recorded at 10 min/frame rate.

File name: Supplementary Movie 5
Description: Basic segregation of zebrafish ectoderm (red) and mesoderm (green) cells in suspension. Epifluorescent microscopy time-lapse video recorded at 10 min/frame rate.

File name: Supplementary Movie 6
Description: Segregation of primary goldfish keratinocytes (PFK, red) and EPC fish keratinocytes (green) in suspension co-culture. Left: untreated co-culture (Ctrl), right: co-culture treated with Y27632 ROCK inhibitor. Time-lapse 3D reconstruction from optical sectioning by structured illumination microscopy, images recorded at 10 min/frame rate.

File Name: Supplementary Movie 7
Description: Segregation of A431 human epithelial carcinoma (red) and HT1080 human fibrosarcoma (green) cells in suspension co-culture. Left: untreated co-culture (Ctrl), right: co-culture treated with Y27632 ROCK inhibitor. Time-lapse 3D reconstruction from optical sectioning by structured illumination microscopy, images recorded at 10 min/frame rate.

File name: Supplementary Movie 8
Description: Segregation of zebrafish ectoderm (red) and mesoderm (green) cells in suspension co-culture. Left: untreated co-culture (Ctrl), right: co-culture treated with Y27632 ROCK inhibitor. Time-lapse 3D reconstruction from optical sectioning by structured illumination microscopy, images recorded at 10 min/frame rate.

File name: Supplementary Movie 9
Description: Aggregation of A431 human epithelial carcinoma cells in suspension culture. Left: A431 cells expressing inactive mutant S100A4 (A431-ctrl), right: A431 cells expressing NM2 assembly inhibitor S100A4 (A431-S100A4). Phase contrast microscopy, time-lapse video recorded at 10 min/frame rate.

File name: Supplementary Movie 10
Description: Segregation of A431 human epithelial carcinoma (A431-ctrl, green) and HT1080 human fibrosarcoma (red) cells in suspension co-culture. Time-lapse 3D reconstruction by structured illumination

microscopy (left) and simultaneous epifluorescent microscopy (right), images recorded at 10 min/frame rate.

File name: Supplementary Movie 11
Description: Segregation of A431 human epithelial carcinoma cells expressing the NM2 assembly inhibitor S100A4 (A431-S100A4, green) and HT1080 human fibrosarcoma cells (red) in suspension co culture. Note the inverted segregation pattern with HT1080 cells segregating to the inside. Time-lapse 3D reconstruction by structured illumination microscopy (left) and simultaneous epifluorescent microscopy (right), images recorded at 10 min/frame rate.

File name: Supplementary Movie 12
Description: Aggregation of zebrafish ectoderm cells in suspension culture. Left: untreated ectoderm cells (ectoderm-ctrl), right: ectoderms cells expressing constitutively active Rho kinase (ectodermcaROCK). Phase contrast microscopy, time-lapse video recorded at 10 min/frame rate.

File name: Supplementary Movie 13
Description: Segregation of zebrafish normal ectoderm (ectoderm-ctrl, red) and mesoderm (green) cells in suspension co-culture. Time-lapse 3D reconstruction by structured illumination microscopy (left) and simultaneous epifluorescent microscopy (right), images recorded at 10 min/frame rate.

File name: Supplementary Movie 14
Description: Segregation of zebrafish ectoderm cells expressing constitutively active Rho kinase (ectoderm-caROCK, red) and mesoderm cells (green) in suspension co-culture. Note the inverted segregation pattern with mesoderm cells segregating to the inside. Time-lapse 3D reconstruction by structured illumination microscopy (left) and simultaneous epifluorescent microscopy (right), images recorded at 10 min/frame rate.

File name: Supplementary Data 1
Description: Source data underlying the graphs presented in Figure 1.

File name: Supplementary Data 2
Description: Source data underlying the graphs presented in Figure 3.

File name: Supplementary Data 3
Description: Source data underlying the graphs presented in Figure 4 and Figure 8.

File name: Supplementary Data 4
Description: Source data underlying the graphs presented in Figure 5 and Figure 9.

File name: Supplementary Data 5
Description: Source data underlying the graphs presented in Figure 7.
