# Supplementary material for: 3D cell segregation geometry and dynamics are governed by tissue surface tension regulation
Source: Commun Biol. 2023 Aug 4;6:817. doi: 10.1038/s42003-023-05181-7 (PMC10403547; doi:10.1038/s42003-023-05181-7)
Supplement: Supplementary file 23 — Reporting Summary [file 42003_2023_5181_MOESM23_ESM.pdf]

Corresponding author(s): Tamás Vicsek

Last updated by author(s): Jul 12, 2023

Reporting Summary

Nature Portfolio wishes to improve the reproducibility of the work that we publish. This form provides structure for consistency and transparency in reporting. For further information on Nature Portfolio policies, see our Editorial Policies and the Editorial Policy Checklist.

Statistics

For all statistical analyses, confirm that the following items are present in the figure legend, table legend, main text, or Methods section.

n/a	Confirmed	
☐	☒	The exact sample size (*n*) for each experimental group/condition, given as a discrete number and unit of measurement
☐	☒	A statement on whether measurements were taken from distinct samples or whether the same sample was measured repeatedly
☐	☒	The statistical test(s) used AND whether they are one- or two-sided<br>*Only common tests should be described solely by name; describe more complex techniques in the Methods section.*
☒	☐	A description of all covariates tested
☒	☐	A description of any assumptions or corrections, such as tests of normality and adjustment for multiple comparisons
☒	☐	A full description of the statistical parameters including central tendency (e.g. means) or other basic estimates (e.g. regression coefficient) AND variation (e.g. standard deviation) or associated estimates of uncertainty (e.g. confidence intervals)
☒	☐	For null hypothesis testing, the test statistic (e.g. F, t, r) with confidence intervals, effect sizes, degrees of freedom and P value noted<br>*Give P values as exact values whenever suitable.*
☒	☐	For Bayesian analysis, information on the choice of priors and Markov chain Monte Carlo settings
☒	☐	For hierarchical and complex designs, identification of the appropriate level for tests and full reporting of outcomes
☒	☐	Estimates of effect sizes (e.g. Cohen's *d*, Pearson's *r*), indicating how they were calculated

Our web collection on statistics for biologists contains articles on many of the points above.

Software and code

Policy information about availability of computer code

Data collection	Image acquisition on a Zeiss Axio Observer Z1 microscope was controlled by commercial AxioVision software (version 4.8) issued by Carl Zeiss Microscopy, LLC.
Data analysis	Microscopic images were analyzed using ImageJ software (version 1.48v) and FIJI software (version 1.50g) issued by National Institutes of Health, USA. Analysis of certain image parameters were carried out using custom-made plugins for NIH ImageJ software that are freely available at GitHub (https://github.com/gulyasmarton/ContractilityAnalyzer.git).

For manuscripts utilizing custom algorithms or software that are central to the research but not yet described in published literature, software must be made available to editors and reviewers. We strongly encourage code deposition in a community repository (e.g. GitHub). See the Nature Portfolio guidelines for submitting code & software for further information.

Data

Policy information about availability of data

All manuscripts must include a data availability statement. This statement should provide the following information, where applicable:

- Accession codes, unique identifiers, or web links for publicly available datasets
- A description of any restrictions on data availability
- For clinical datasets or third party data, please ensure that the statement adheres to our policy

All data supporting the findings of this study are included in this published article and its Supplementary Information and Supplementary Data files.

Human research participants

Policy information about studies involving human research participants and Sex and Gender in Research.

Reporting on sex and gender	*Use the terms sex (biological attribute) and gender (shaped by social and cultural circumstances) carefully in order to avoid confusing both terms. Indicate if findings apply to only one sex or gender; describe whether sex and gender were considered in study design whether sex and/or gender was determined based on self-reporting or assigned and methods used. Provide in the source data disaggregated sex and gender data where this information has been collected, and consent has been obtained for sharing of individual-level data; provide overall numbers in this Reporting Summary. Please state if this information has not been collected. Report sex- and gender-based analyses where performed, justify reasons for lack of sex- and gender-based analysis.*
Population characteristics	*Describe the covariate-relevant population characteristics of the human research participants (e.g. age, genotypic information, past and current diagnosis and treatment categories). If you filled out the behavioural & social sciences study design questions and have nothing to add here, write "See above."*
Recruitment	*Describe how participants were recruited. Outline any potential self-selection bias or other biases that may be present and how these are likely to impact results.*
Ethics oversight	*Identify the organization(s) that approved the study protocol.*

Note that full information on the approval of the study protocol must also be provided in the manuscript.

Field-specific reporting

Please select the one below that is the best fit for your research. If you are not sure, read the appropriate sections before making your selection.

☒ Life sciences ☐ Behavioural & social sciences ☐ Ecological, evolutionary & environmental sciences

For a reference copy of the document with all sections, see nature.com/documents/nr-reporting-summary-flat.pdf

Life sciences study design

All studies must disclose on these points even when the disclosure is negative.

Sample size	No calculations to predetermine sample sizes were used generally. For cell contact angle calculations n>160 cell doublets (sample unit) were analyzed for each group and two-tailed Student's t-test was made (p = 2.38x10^-11). For the analysis of homotypic contact strength in cell doublets, the sample sizes (n>12 for each group) were determined on the basis of previous published work with other cell types. For the analysis of time-dependent growth of homotypic cell cluster sizes during 3D segregation in cell suspension samples (each containing n>3 000 cells), the number of independent samples analyzed was in the range of 6 to 17, representing independent samples from at least 3 independent sets of experiments, determined on the basis of previous published work. For the calculation mean cluster size value in one 3D cell culture (i.e. one independent sample), in one z-plane, at one time point, n>1000 data points were analyzed in the corresponding image of that sample.
Data exclusions	No data were excluded from analysis.
Replication	All attempts on replication were successful.
Randomization	*Describe how samples/organisms/participants were allocated into experimental groups. If allocation was not random, describe how covariates were controlled OR if this is not relevant to your study, explain why.*
Blinding	*Describe whether the investigators were blinded to group allocation during data collection and/or analysis. If blinding was not possible, describe why OR explain why blinding was not relevant to your study.*

Reporting for specific materials, systems and methods

We require information from authors about some types of materials, experimental systems and methods used in many studies. Here, indicate whether each material, system or method listed is relevant to your study. If you are not sure if a list item applies to your research, read the appropriate section before selecting a response.

Materials & experimental systems

n/a	Involved in the study	
☐	☒	Antibodies
☐	☒	Eukaryotic cell lines
☒	☐	Palaeontology and archaeology
☐	☒	Animals and other organisms
☒	☐	Clinical data
☒	☐	Dual use research of concern

Methods

n/a	Involved in the study	
☒	☐	ChIP-seq
☒	☐	Flow cytometry
☒	☐	MRI-based neuroimaging

Antibodies

Antibodies used	Primary antibody: Anti-MYL12A phospho S19 primary antibody against phosphorylated (pS19) myosin light chain, developed: in rabbit, clonality: polyclonal, manufacturer: Abcam, catalogue number: ab2480;<br>Secondary antibody: Anti-rabbit secondary antibody conjugated with AlexaFluor-555, developed: in goat, manufacturer: Southern Biotech, catalogue number: 4030-32.
Validation	Abcam ab2480, Anti-MYL12A phospho S19 primary antibody against phosphorylated (pS19) myosin light chain.<br>Manufacturer's statement at product website (https://www.abcam.com/products/primary-antibodies/myl12a-phospho-s19-antibody-ab2480.html)<br>Specificity<br>ab2480 can detect the phosphorylated serine residue in both the human and mouse protein. In human the phosphorylation site is pS19, while in mouse the site maps to pS20.<br>This phospho specific polyclonal antibody is specific for phosphorylated pS19 of human myosin light chain. Reactivity with non-phosphorylated human myosin light chain is less than 1% by ELISA. The selected peptide sequence used to generate the polyclonal antibody is located near the amino terminal end of the polypeptide corresponding to the smooth/non-muscle form of myosin regulatory light chain found in cardiac myocytes in addition to smooth and non-muscle cells. This sequence differs from that of the sarcomeric/cardiac form of myosin regulatory light chain that has a different sequence around the phosphorylation site. BLAST search analysis was used to determine that the smooth and non-muscle forms of myosin regulatory light chain have identical sequences.<br>Tested applications<br>Suitable for: ELISA, ICC/IF, IHC-FoFr, WB, IHC-P, IP<br>Species reactivity<br>Reacts with: Mouse, Rat, Human, Caenorhabditis elegans, Recombinant fragment

Eukaryotic cell lines

Policy information about cell lines and Sex and Gender in Research

Cell line source(s)	EPC fish keratinocyte cell line was purchased from ATCC (CRL-2872). Primary fish keratinocytes were isolated from scales of goldfish (Carassius auratus). A431 human epithelial carcinoma cell line (CRL-1555) and HT1080 human fibrocarcinoma cell line (CCL-121) were obtained from ATCC. Germ layer progenitor cell types were isolated from zebrafish (Danio rerio) embryos.
Authentication	None of the cell lines used were authenticated in other ways than by the commercial suppliers specified herein.
Mycoplasma contamination	The commercial cell lines were not tested for mycoplasma contamination.
Commonly misidentified lines<br>(See ICLAC register)	*Name any commonly misidentified cell lines used in the study and provide a rationale for their use.*

Animals and other research organisms

Policy information about studies involving animals; ARRIVE guidelines recommended for reporting animal research, and Sex and Gender in Research

Laboratory animals	Goldfish (Carassius auratus) of 1 year age were used (but not sacrificed) in this study to isolate keratinocytes from scales. Embryos from zebrafish (Danio rerio) were used (sacrificed) in this study for isolation of germ layer progenitor cells.
Wild animals	The study did not involve wild animals.
Reporting on sex	Information pertaining to sex has not been collected.
Field-collected samples	The study did not involve samples collected from the field.
Ethics oversight	Study protocols for zebrafish (Danio rerio) used in this study were approved by the Hungarian National Food Chain Safety Office (Permit Number: XIV-I-001/515-4/2012). Conditions of the use of goldfish (Carassius auratus) were approved by University Institutional Animal Care and Use Committee of ELTE Eötvös Loránd University, Budapest, Hungary.

Note that full information on the approval of the study protocol must also be provided in the manuscript.
